# High-Frame Rate Vector Flow Imaging Technique: Initial Application in Evaluating the Hemodynamic Changes of Carotid Stenosis Caused by Atherosclerosis

**DOI:** 10.3389/fcvm.2021.617391

**Published:** 2021-03-08

**Authors:** Yijie Qiu, Yi Dong, Feng Mao, Qi Zhang, Daohui Yang, Kailing Chen, Shuainan Shi, Dan Zuo, Xiaofan Tian, Lingyun Yu, Wen-Ping Wang

**Affiliations:** ^1^Department of Ultrasound, Zhongshan Hospital, Fudan University, Shanghai, China; ^2^Shanghai Institute of Medical Imaging, Zhongshan Hospital, Fudan University, Shanghai, China

**Keywords:** vector flow imaging (V Flow), wall shear stress, hemodynamic change, carotid atherosclerotic plaques, stenosis

## Abstract

**Objective:** To investigate the value of high-frame rate vector flow imaging technique (V flow) in evaluating the hemodynamic changes of carotid stenosis caused by atherosclerotic plaques.

**Methods and Materials:** In this prospective study, patients with stenosis rate (diameter) ≥30% caused by carotid atherosclerotic plaques were included. Degrees of carotid stenosis were graded according to North American Symptomatic Carotid Endarterectomy Trial criteria: moderate (30–69%) or severe (70–99%). Mindray Resona 7s ultrasound machine with a linear array transducer (3–11 MHz) was used for ultrasound examinations. The mean WSS value of carotid arteries was measured at the proximal, narrowest region and distal of carotid stenosis. The mean WSS values were correlated with peak systolic velocity (PSV) measured by color Doppler flow imaging and stenosis degree detected by digital subtraction angiography (DSA). The vector arrows and flow streamline detected by V flow dynamic imaging were analyzed. Imaging findings of DSA in carotid arteries were used as the gold standard.

**Results:** Finally, 51 patients were included. V flow measurements were performed successfully in 17 patients (100%) with moderate-grade stenosis and in 30 patients (88.2%) with severe-grade stenosis. Dynamic V flow imaging showed yellow or red vectors at the stenotic segment, indicating fast speed blood flow (up to 260.92 cm/s). Changes of streamlines were detected in the stenotic segment. The mean WSS value measured at the narrowest region of the carotid artery had a moderately positive correlation with stenosis degree (*r* = 0.58, *P* < 0.05) and PSV value (*r* = 0.54, *P* < 0.05), respectively. Significant difference was detected in mean WSS value at the narrowest region of the carotid artery between severe carotid stenosis (1.47 ± 0.97 Pa) and moderate carotid stenosis (0.96 ± 0.44 Pa) (*P* < 0.05).

**Conclusion:** The hemodynamic changes detected by V flow of the carotid stenosis might be a potential non-invasive imaging tool for assessing the degree of carotid stenosis.

## Introduction

Carotid stenosis caused by atherosclerotic plaque is the main cause of ischemic stroke and has a high incidence rate among people 65 years or older (1, 2). Preoperative diagnosis of carotid artery stenosis is important for the further choice of treatment as a reasonable treatment method should be selected according to the patient's carotid stenosis degree. Asymptomatic patients with severe carotid artery stenosis or patients with symptomatic moderate carotid artery stenosis require surgical treatment (3, 4). However, currently, the diagnostic sensitivity of carotid stenosis by imaging modalities varies from 31 to 85%, and the specificity varies from 54 to 85% (5). Hemodynamic change, the functional evaluation, is also one of the crucial factors in evaluating carotid stenosis. Combined functional evaluation such as hemodynamic changes (including the blood flow velocity, flow direction, flow pattern, and blood viscosity) of the carotid artery may provide additional information for the diagnosis and decision-making process of the carotid stenosis.

Wall shear stress (WSS) is the frictional force exerted on the endothelial surface of the vessel wall, which is strongly influenced by hemodynamic changes (6, 7). It has been reported to play an important role in the development of carotid plaque. Low and/or oscillatory WSS value affects the morphology, structure, and function of endothelial cells of vessel wall (8, 9). High WSS value promotes the high-risk plaque, rupture of plaque (10, 11). To obtain the WSS value preoperatively is of vital importance for further clinical decision-making process of carotid stenosis. Currently, there are some medical imaging methods that can be used for WSS measurement, including phase contrast magnetic resonance imaging (PC-MRI), computed tomography angiography (CTA), and ultrasound. PC-MRI is the most commonly used MRI methods to measure the WSS value with high tissue resolution (12). However, its disadvantages include low temporal and spatial resolution and long measurement time. Only few studies used CTA to calculate the WSS value; meanwhile, it has the potential of radiation exposure and limitation for analyzing plaque components. Depending on the unique advantages such as convenience, non-radiation, and effectiveness, conventional ultrasound is also the frequently used tool to measure the WSS value.

High-frame rate vector flow imaging technique (V flow) based on ultrasound is an emerging quantitative imaging method for superficial vessels, mainly focused on carotid artery (13). WSS value is the quantitative parameter under the V flow module. Real-time high frame rate V flow has been reported to be able to depict the blood flow characteristics in the carotid artery intuitively, especially in complicated carotid stenosis circumstance (14).

The aim of our study is to investigate the value of V flow in evaluating the hemodynamic changes of carotid stenosis caused by atherosclerotic plaques.

## Patients and Methods

### Patients

This prospective study was approved by the Ethics Committee of Zhongshan Hospital, Fudan University (approval no. B2019-295R), and informed consent was obtained in all patients. The inclusion criteria were as following: (1) patients' aging from 18 to 80 years old; (2) carotid stenosis rate (diameter) ≥30% measured by digital subtraction angiography (DSA); (3) carotid artery stenosis in only one side; and (4) patients planned to accept carotid artery stenting or carotid endarterectomy in our hospital. Exclusion criteria included (1) carotid completely occlusion patients; (2) patients with obvious heart, liver, and kidney dysfunctions; (3) patients with previous carotid artery stenting or carotid endarterectomy history; and (4) carotid artery could not be observed clearly on B-mode ultrasound (BMUS).

The stenosis degrees of the carotid artery measured by DSA were graded according to the North American Symptomatic Carotid Endarterectomy Trial (NASCET) criteria: moderate (30–69%) or severe (70–99%) (15).

### Ultrasound Examination of Carotid Artery

All ultrasound examinations were performed with a Mindray Resona 7s ultrasound machine (Shenzhen Mindray Bio-Medical Electronic Co., Shenzhen, China) equipped with a linear array transducer (3–11 MHz). The machine was equipped with updated V flow software for carotid artery analysis.

Ultrasound examinations were performed 1 day before carotid artery stenting or carotid endarterectomy surgery. During ultrasound examination, patients were lying on supine positions with the posterior neck supported by a thin pillow. First, BMUS scans were obtained in the side of carotid artery stenosis in longitudinal and transverse planes, from the initial segment of common carotid artery to the common carotid bifurcation and then to internal and external carotid artery. The plaque echogenicity was observed and documented. Meanwhile, the peak systolic velocity (PSV) value was measured at the narrowest region of the carotid artery by color Doppler flow imaging (CDFI) for three times to obtain the average value.

**Figure 1:**
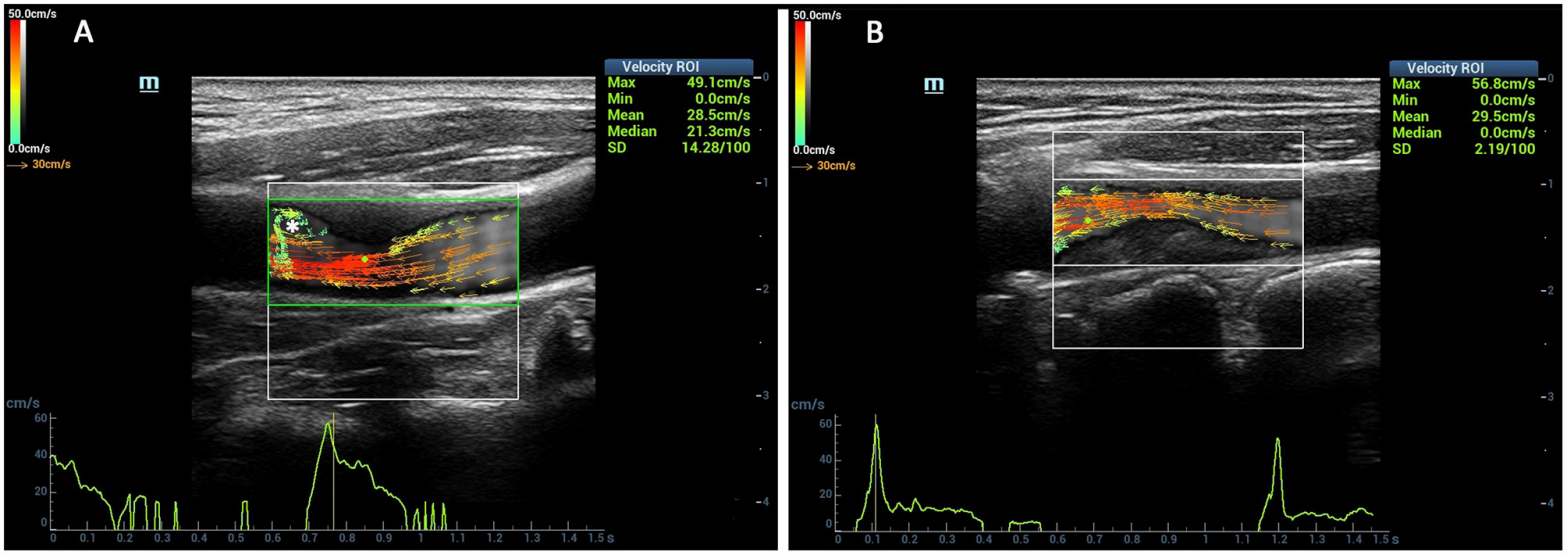
Comparison of the vector arrows between stenotic segment and normal carotid artery. The red vectors mean the high velocities, yellow and orange vectors mean medium velocities, and green vectors mean low velocities. The vector arrows in the carotid artery lumen changed from yellow to red color at the stenotic segment **(A,B)**. The complex flow was detected at the distal of the stenosis (asterisk) **(A)**.

V flow measurement settings included the following: the frequency is 5.0 MHz, the depth is 2 to 4 cm, the arrow life cycle is 25 ms, and the arrow density is 10%. After obtained clear visualization of carotid stenosis, the “Update” button was pressed to acquire dynamic V flow images. In V flow mode, the blood flow signals were represented by various dynamic vector arrows. Flow velocity was represented by different color and length of the vector arrows. The red vectors mean the high velocities, yellow and orange vectors mean medium velocities, and green vectors mean low velocities (Figure 1). For the length of the vector arrows, the longer the arrows, the faster the flow velocity. Meanwhile, streamlines represent the overall flow of blood (Figure 1). The mean WSS values were measured automatically by adjusting the reference line to coincide with the vascular wall at the peak of systole during the detected cardiac cycle. The WSS value of V flow as a function of time can be estimated by the following:

(1) $$\begin{array}{r} \underset{\tau }{\to }(t)=\mu \overset{i=N}{\underset{i=1}{\sum^{\text{​}}}}\frac{\underset{w}{\to }.\underset{v_{i}}{\to }(t)}{\Delta r_{i}} \end{array}$$

where μ is blood viscosity; $\vec{w}$ denotes the direction of $\vec{\tau }$ and is a unit vector; $\vec{v_{i}}$ is the vector velocity; Δ*r*_*i*_ and is the distance between the *i*th velocity measurement and the WSS measurement location (16). One radiologist with 5 years of carotid ultrasound experience performed V flow scan, who was blind to the patient's clinical data and final pathological findings. The entire process for V flow measurements, from acquisition of the dynamic V flow images to WSS measurements, takes about 3 min 20 s for each carotid artery (Table 1).

**Table 1:** The V Flow imaging flow chart and the time consumed for wall shear stress (WSS) measurements (s).

**Acquisition of dynamic V flow images**	**Automatic imaging reprocessing**	**Video clip playback**	**Velocity measurements**	WSS measurements1^*^
1.5	≈30	≈40–60	≈20	≈90

* *Six series of three measurements*.

### DSA Measurement

DSA was performed in each patient included in our study. Imaging results of DSA during the surgery were used as the gold standard. With NASCET criteria, measuring stenosis is taking the inner diameter of the distal normal lumen as the basic inner diameter (A), and the inner diameter of residual lumen at stenosis segment as the measurement (B). And the degree of carotid stenosis = (1–B/A) × 100% (15).

### Statistical Analysis

Continuous variables were expressed as mean ± standard deviation (SD). The relationship between mean WSS value and PSV and that between mean WSS value and carotid artery stenosis degree were calculated by Pearson correlation coefficient. The difference of mean WSS value between moderate and severe carotid stenoses was performed using independent-samples *t*-test. All data were calculated using the software program SPSS Statistics (version 22.0, IBM, Armonk, NY, USA) and GraphPad Prism (version 7, GraphPad Software, Inc.). *P* < 0.05 was considered statistically.

## Results

### Patients Characteristics

**Figure 2:**
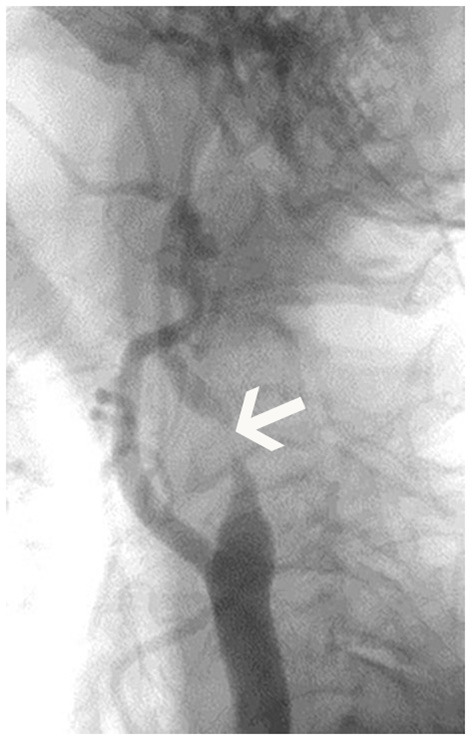
Digital subtraction angiography (DSA) imaging of patient. The figure displayed the severe carotid stenosis (arrow) in the left internal carotid artery.

Between June 2019 and August 2020, 51 patients were finally included. There were three females and 48 males in the study group, and the mean age was 66.1 ± 5.4 years. Twenty-seven carotid stenoses were in the right carotid artery, and 24 carotid stenoses were in the left side (Table 2). Surgery included carotid artery stenting (*n* = 42) or carotid endarterectomy (*n* = 9). According to DSA results (Figure 2), patients were divided into severe grade of carotid stenosis (*n* = 34) and moderate grade of carotid stenosis (*n* = 17) (Figure 2).

**Table 2:** The baseline data of stenotic carotid artery patients.

	**Value or number**
Age (year)	66.1 ± 5.4
**Sex**	
Female	3 (5.88%)
Male	48 (94.12%)
**Side of the carotid artery**	
Right	27 (52.94%)
Left	24 (47.06%)
**History**	
Hypertension	31 (60.78%)
Diabetes	15 (29.41%)
Smoke	19 (37.25%)
Hyperlipidemia	25 (49.02%)
**Surgery**	
Carotid artery stenting	42 (82.35%)
Carotid endarterectomy	9 (17.64%)
**DSA findings**	
Moderate carotid stenosis	17 (33.33%)
Severe carotid stenosis	34 (66.67%)

*Data are shown as the number(percentage) or frequency ± standard errors as appropriate*.

### B Mode Ultrasound Features

Most patients (*n* = 47, 92.16%) have stenosis in the internal carotid artery and carotid bifurcation in our study. Among all carotid plaques, 25 (49.02%) were hypoechoic, 19 (37.25%) were mixed echoic, 4 (7.84%) were isoechoic, and 3 (5.88%) were hyperechoic. For morphology features detected by BMUS, eight plaques had lipid necrotic core, 19 plaques had calcifications, and seven plaques had ulcer in this study.

The mean value of resistance index in 51 patients was 0.70 (Table 3).

**Table 3:** B mode ultrasound features of the carotid stenosis.

	**Value or number**
**Location**	
Internal carotid artery	40 (78.43 %)
Carotid bifurcation	7 (13.73%)
Common carotid artery	4 (7.84%)
**Echogenicity of the plaque**	
Hypoechoic	25 (49.02%)
Isoechoic	4 (7.84%)
Hyperechoic	3 (5.88%)
Mixed echoic	19 (37.25%)
**Morphology features**	
Lipid necrotic core	8 (15.69%)
Calcification	19 (37.25%)
Ulcer	7 (13.73%)
Resistance index (RI)	0.70 ± 0.10
**Grade of carotid stenosis (diameter)**	
Moderate (30–69%)	17 (33.33%)
Severe (70–99%)	34 (66.67%)
Peak systolic velocity (cm/s)	214.75 ± 115.12

*Data are shown as the number(percentage) or frequency ± standard errors as appropriate*.

While using CDFI, the average value of PSV measured at the narrowest location of the carotid artery was 214.75 ± 115.12 cm/s (Table 3).

### V Flow Measurement

Fifty-one inpatients underwent V flow measurement 1 day before the surgery. V flow measurements were performed successfully in 100% (17/17) of moderate carotid stenosis patients and in 88.2% (30/34) of patients with severe carotid stenosis (*P* > 0.05).

**Figure 3:**
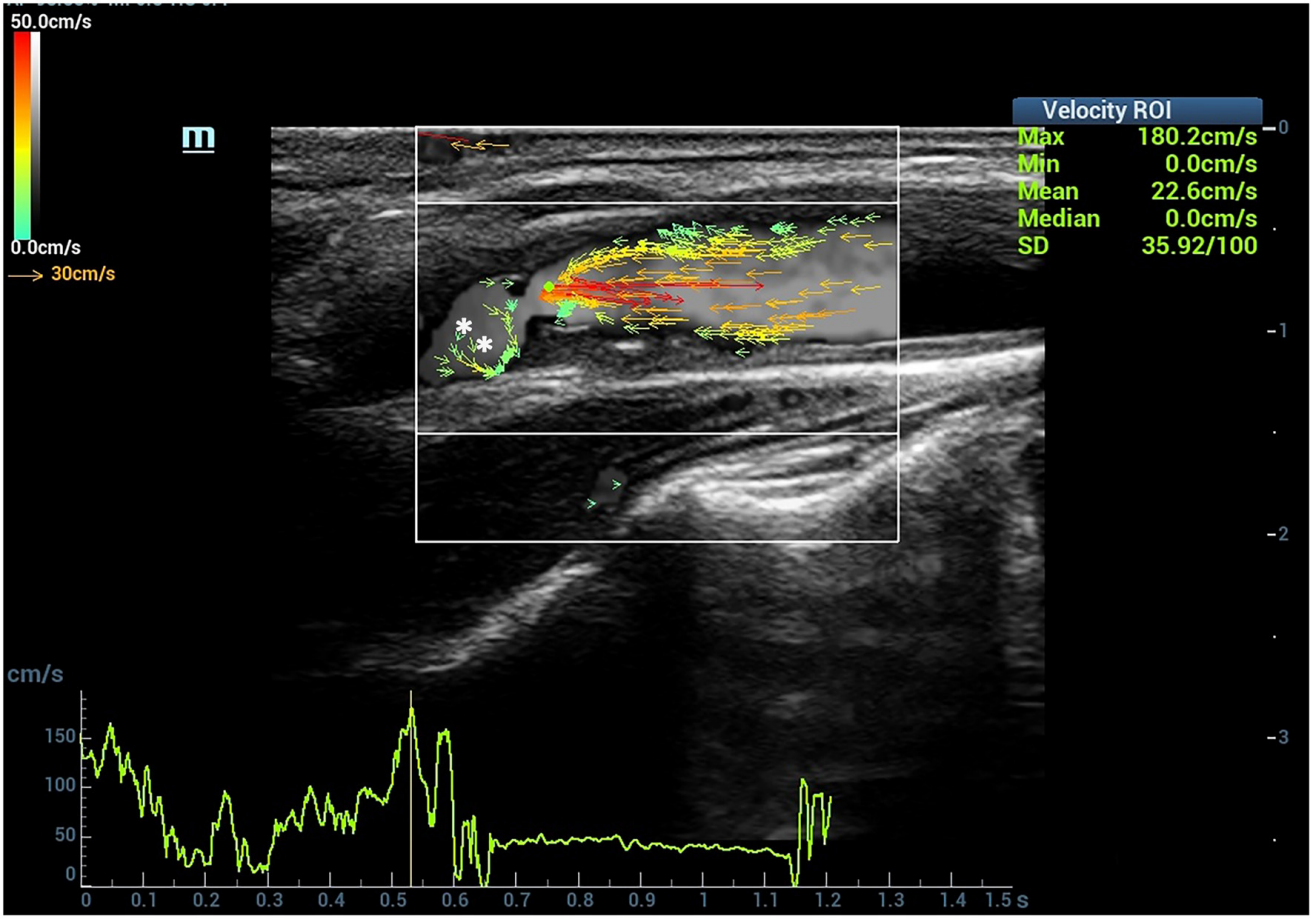
Complex blood flow in the patients with carotid stenosis. Vortex (short green or yellow vectors) (asterisk) can be detected in the distal of carotid stenosis.

The length of the blood flow vector arrows became longer at the stenotic segment than the surrounding vessel wall. Meanwhile, the most common colors of the vector arrows were yellow or red at the stenotic segment. Red vectors shown by dynamic V flow imaging indicated fast speed blood flow (180.2 cm/s) at the stenotic segment in moderate- to severe-grade carotid stenosis patients (Figure 3). The streamlines were observed to be changed at the stenotic segment caused by carotid atherosclerosis plaques (Figure 1). Complex disturbed blood flow, for example, vortex, can be intuitively displayed in the distal of the carotid artery to stenosis (Figure 3).

### V Flow Parameters

**Figure 4:**
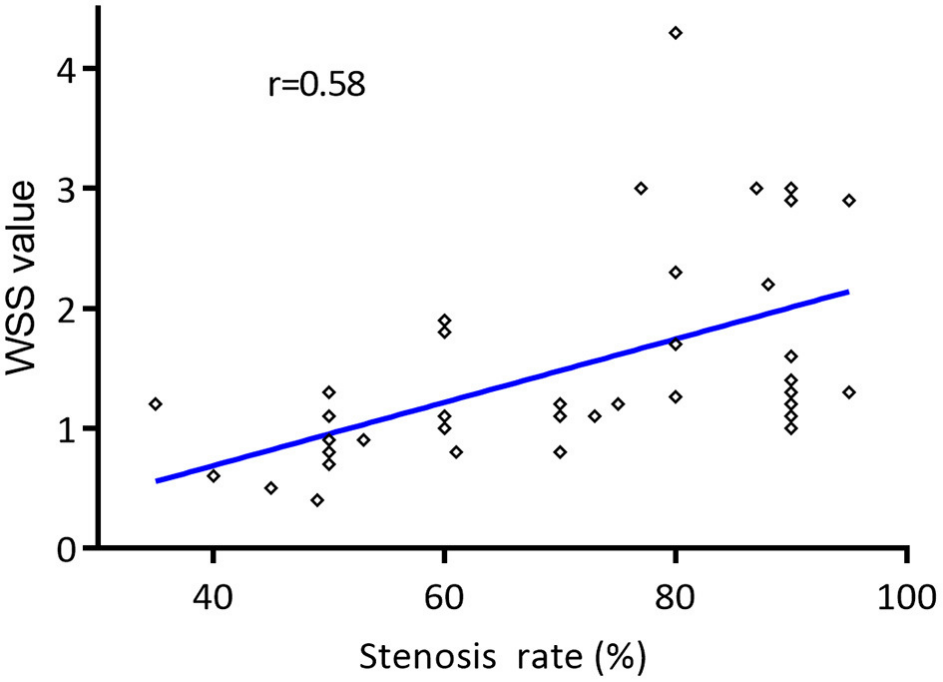
Correlation of the mean wall shear stress (WSS) values of narrowest region of the carotid artery and the degree of carotid stenosis. The mean WSS value of the narrowest region of the carotid artery had a moderately positive correlation with the degree of carotid stenosis by Pearson correlation coefficient (*r* = 0.58, *P* < 0.05).

The mean WSS value of the narrowest region of the carotid artery had a moderately positive correlation with stenosis rate (*r* = 0.58, *P* < 0.05) (Figure 4). Meanwhile, moderately positive correlation was reached between mean WSS value at the narrowest region of the carotid artery and PSV value measured in the narrowest region (*r* = 0.54, *P* < 0.05). In addition, the mean WSS value in the narrowest region of severe carotid stenosis (1.47 ± 0.97 Pa) was significantly higher than the moderate carotid stenosis (0.96 ± 0.44 Pa) (*P* < 0.05). No significant difference of mean WSS value was detected in the proximal and distal of carotid stenosis between the moderate and severe carotid stenoses (*P* > 0.05) (Table 4).

**Table 4:** The mean WSS value for different degrees of carotid stenosis (*n* = 51).

**Grade of carotid stenosis according to NASCET criteria (15)**	**Proximal of stenosis (Pa)**	**Narrowest region of the carotid artery (Pa)**	**Distal of stenosis (Pa)**
Moderate (30–69%)	0.72 ± 0.33	0.96 ± 0.44	0.63 ± 0.38
Severe (70–99%)	0.79 ± 0.62	1.47 ± 0.97	0.67 ± 0.52
	*P* > 0.05	*P* < 0.05	*P* > 0.05

*The mean WSS value of the proximal, narrowest region and distal of the stenosis was compared between the moderate and the severe carotid stenosis*.

*WSS, Wall shear stress; NASCET, North American Symptomatic Carotid Endarterectomy Trial*.

The mean WSS values were higher at the narrowest region and proximal of stenosis than the distal of stenosis (Table 4). In the severe carotid stenosis, seven patients had PSV value inconsistent with the degree of stenosis. Exclude these seven patients and calculate the WSS value of the severe stenosis group again. The mean WSS value in proximal of stenosis (0.89 ± 0.65 pa) and narrowest region (1.77 ± 0.87 pa) was still higher than the part of stenosis (0.81 ± 0.60 pa) in severe carotid stenosis.

### Clinical Examples

**Figure 5:**
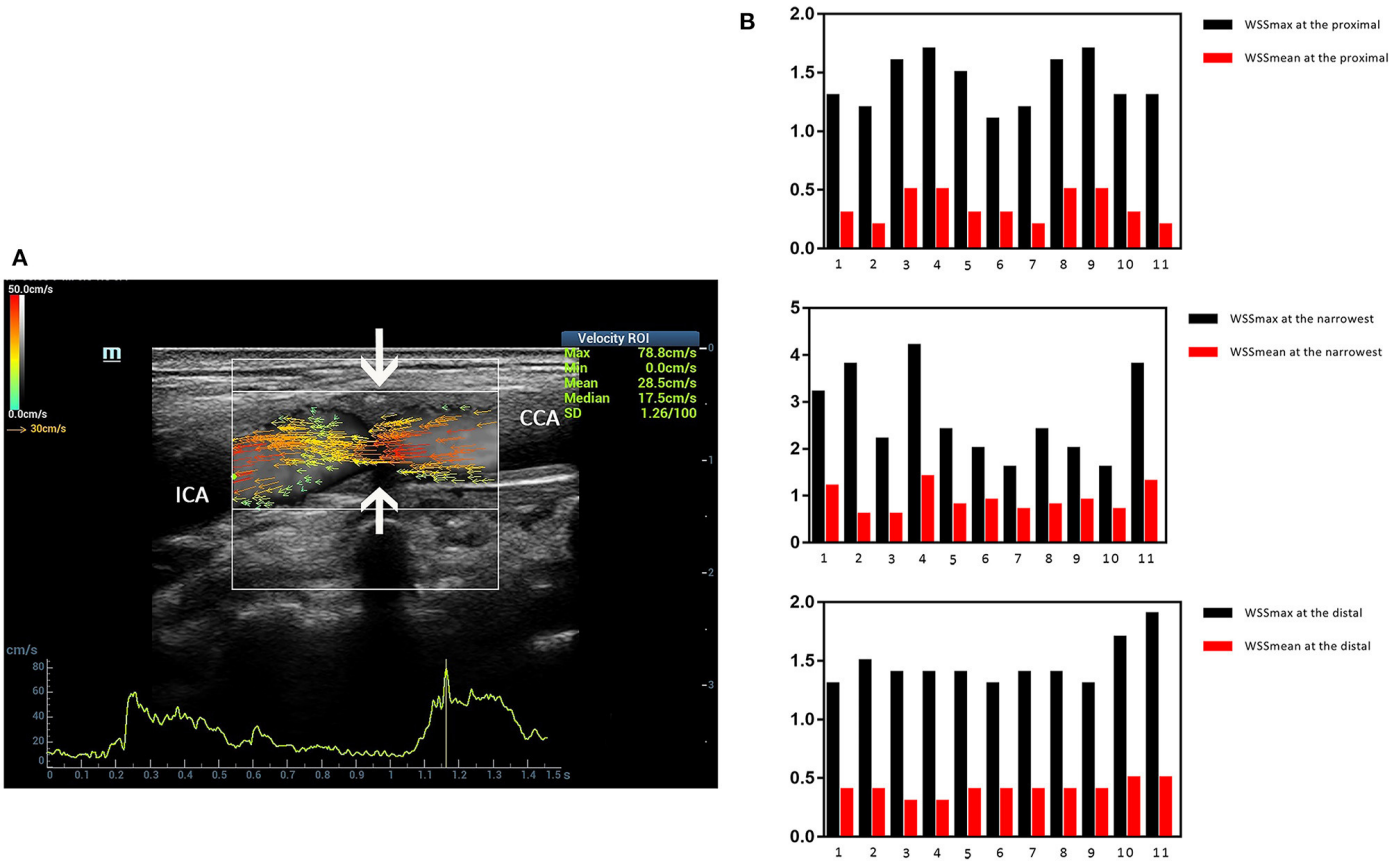
Mild carotid stenosis. V flow imaging shows high-velocity flow (red vectors) moving at the carotid stenosis(arrows) **(A)**. Wall shear stress (WSS) point-by-point measurements at the proximal, distal, and narrowest regions had mean WSS (WSSmean) and maximum WSS (WSSmax) values showing in the figures. Abnormal low WSSmean values (<0.4 Pa) were detected at the proximal (0.2–0.3 Pa) and distal region (0.3 Pa) **(B)**. CCA indicates common carotid artery; and ICA indicates internal carotid artery.

**Figure 6:**
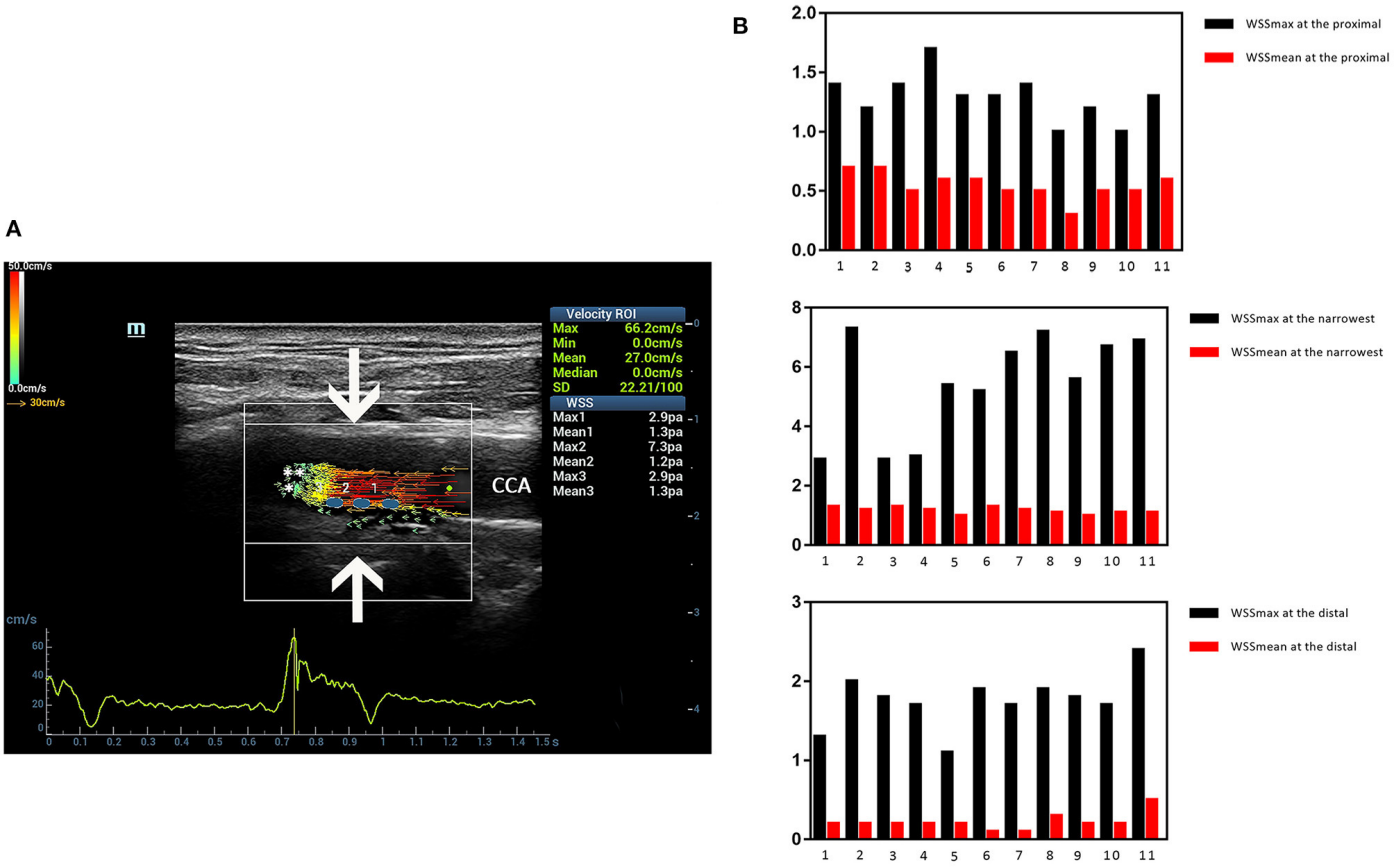
Moderate-grade carotid stenosis. V flow imaging shows high-velocity flow (red vectors) at the stenosis region (arrows), accompanied by complex flow at the distal region (asterisk) **(A)**. Wall shear stress (WSS) point-by-point measurements show a high maximum WSS (WSSmax) value of 7.3 Pa at the narrowest region of the far wall (blue dots on A). Abnormal low mean WSS (WSSmean) values (<0.4 Pa) were detected at the proximal (0.2–0.3 Pa) and distal region (0.3 Pa) **(B)**. CCA indicates common carotid artery.

**Figure 7:**
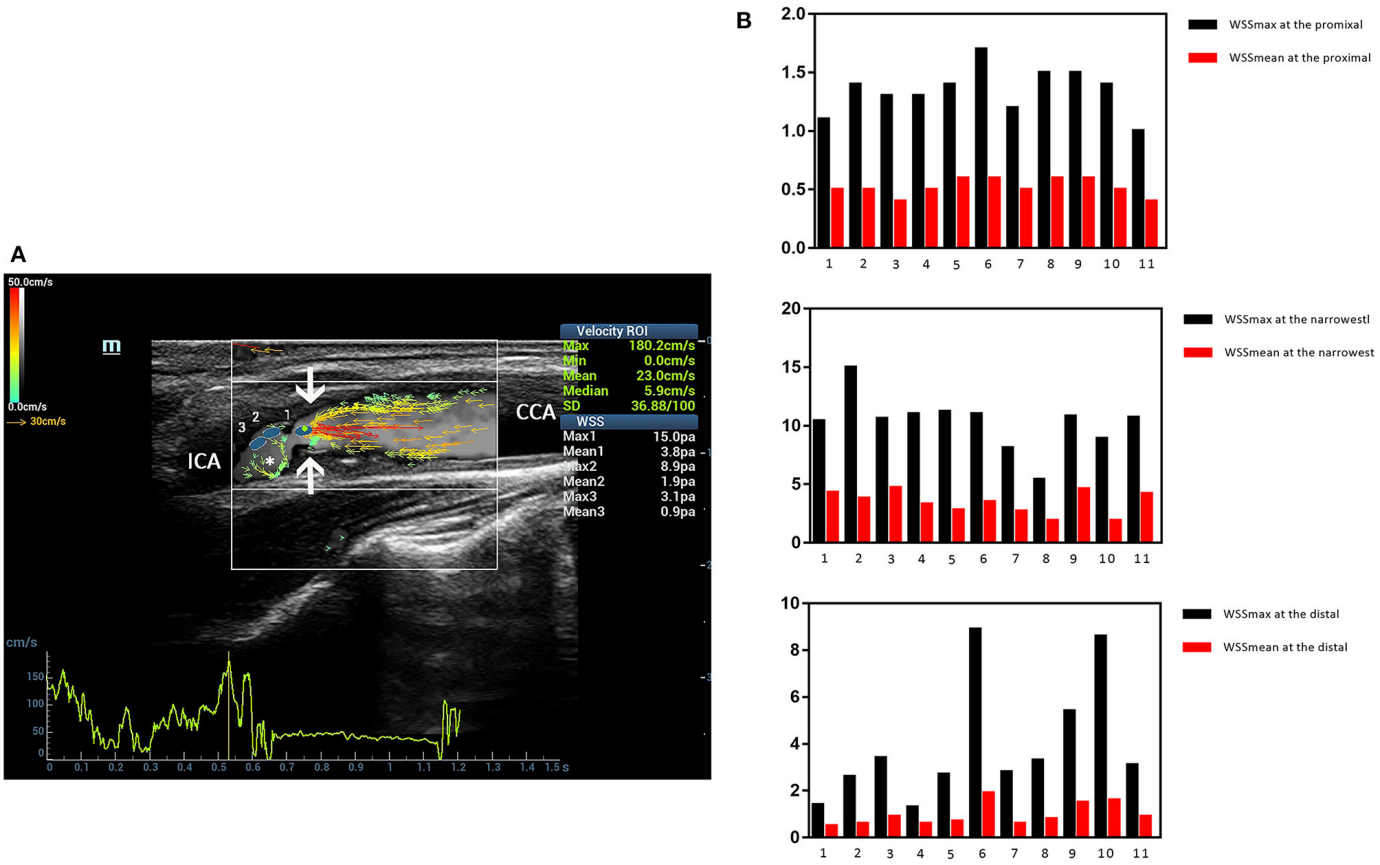
Severe carotid stenosis. V flow imaging shows very high-velocity flow (red vectors) at the narrowest region (arrows) and vortex at the distal of the stenosis (asterisk) **(A)**. Wall shear stress (WSS) point-by-point measurements show a high maximum WSS (WSSmax) value of 15.0 Pa at the narrowest region (blue dot 1 on **A**) **(B)**. CCA indicates common carotid artery, and ICA indicates internal carotid artery.

Three ultrasound images were shown as the clinical examples of the V flow used in the different degree of carotid stenosis (Figures 5–7). V flow imaging all shows high-velocity flow (red vectors) moving at the carotid stenosis (arrows) (Figures 5–7). WSS point-by-point measurements show a high maximum WSS (WSSmax) value at the narrowest region in the moderate and severe carotid stenoses (Figures 6, 7). Abnormal low mean WSS (WSSmean) values (<0.4 Pa) were detected at the proximal and distal of the mild and moderate carotid stenosis (Figures 5, 6).

## Discussion

The current guidelines take degree of carotid stenosis as the main criterion for selecting treatment options. The preoperative diagnosis of degree of carotid stenosis is critical for clinical decision making (3). Besides stenosis degree, the carotid artery hemodynamic changes are the crucial reasons for the formation of carotid atherosclerosis plaques. In addition, preoperative evaluation of carotid artery hemodynamic changes in blood flow velocity, flow direction, streamline, and WSS value is significant for assessing the degree of carotid stenosis. V flow is stable and repeatable in evaluation of blood flow in carotid arteries. Vector flow mapping based on the transverse oscillation can also be used in the visual evaluation of the carotid artery (17). It was reported that the WSS was negatively correlated with age and intima-media thickness, which has potential to be an indicator of atherosclerosis (18). Vector flow imaging method has broad application prospects in the assessment of carotid atherosclerosis.

In our previous work, we reported that different sides of the carotid artery, different locations (initial segment, middle segment, and near-bifurcation segment of the carotid artery) showed no infect on the measurement of WSS values in healthy adults. The interobserver agreement of WSS measurement is excellent (19). V flow imaging method can also be effectively performed in patients with carotid stenosis. In this study, V flow measurements were performed successfully in 100% patients with moderate carotid stenosis and in 88.2%patients with severe carotid stenosis.

V flow adopts the vector projectile imaging method to get the high-frame rate images for dynamic display of the blood flow (14, 20). It can display the blood flow velocity and direction at various points in the blood vessel in real time by vector arrows (14). Normally, the velocity of blood flow close to the carotid vessel wall is slower than the flow near the center of the vessel, and it is common to see the green vector arrows near the vessel wall (21). We evaluated the hemodynamic changes of the patients with carotid stenosis caused by atherosclerosis plaque. The dynamic V flow images showed the flow vectors changed at the stenotic segment. The color of the vector arrows changed from green or yellow to orange or red accompanied by an increase in length of vector arrows at the stenotic segment. In addition, the streamlines also changed in the stenotic segment, which could express the seriousness of the carotid stenosis (14). As the degree of carotid stenosis increases, laminar flow will be disturbed, and the complex distribution of blood flow occurs, such as vortex (22) (Figure 3). V flow can intuitively display the complex disturbed blood flow in carotid stenosis according to their hemodynamic changes (14, 23).

Measurement of WSS value is another unique function of V flow imaging method. With the multidirectional transmissions and receptions methods, the true velocity vectors will be achieved without the correction of angle (24). WSS value analysis will be achieved by setting the measurement reference line parallel to the vessel wall and close to the vessel wall after obtaining dynamic blood flow. The WSS value calculated by V flow is convenient and accurate in evaluating carotid artery function (24, 25). High WSS value was considered to be related with plaque instability (10, 26). Most of the plaque ruptures occur at the narrowest region or the proximal of stenosis (26, 27). In our study, the WSS values measured at the narrowest region and the proximal of stenosis were higher than that in the distal of stenosis in one cardiac cycle. This is consistent with the narrowest region or the proximal of stenosis most likely to rupture and ulcer (28, 29). Therefore, the mean WSS value of the carotid artery measured by V flow may be a potential indicator for predicting plaque rupture.

To our knowledge, this is the first study focused on the WSS value measured by the V flow technology in the evaluation of carotid stenosis. The mean WSS value at the narrowest region of the carotid artery had a moderately positive correlation with stenosis rate (*r* = 0.58, *P* < 0.05). In addition, the mean WSS value at the narrowest region of the carotid artery was significantly higher in severe carotid stenosis (1.47 ± 0.97 Pa) than moderate carotid stenosis (0.96 ± 0.44 Pa) (*P* < 0.05). The WSS measurements at the narrowest region measured by V flow technology have a potential role to distinguish severe carotid stenosis from the moderate ones, which may be helpful for the decision of patient's further treatment plan.

Moderately positive correlation was reached between mean WSS value at the narrowest region of the carotid artery and PSV value measured in the narrowest region (*r* = 0.54, *P* < 0.05) in this study. With the increase in carotid stenosis degree, the PSV value measured at the narrowest region of the carotid artery increases. However, when the carotid stenosis rate is >90%, the PSV of some patients will decline (30), which means that the stenosis rate does not match the PSV value. In our results, there were seven patients whose stenosis rate did not match the PSV value. Excluding these patients, the mean WSS value measured at the narrowest region was even higher (1.77 ± 0.87 Pa), which indicated the risk of the plaque rupture.

In mild carotid stenosis, WSSmax values were within the reference range. However, abnormal low WSSmean values (<0.4 Pa) were detected at the proximal (0.2–0.3 Pa) and distal of the stenosis (0.3 Pa), which indicated the plaque is prone to develop. In moderate carotid stenosis, WSS point-by-point measurements show a high WSSmax value at the narrowest region and low WSSmean values at the proximal and distal of the stenosis. Even higher WSS values (15.0 Pa) have been observed at the narrowest region in the severe carotid stenosis, which is accompanied by vortex or turbulent flow. For the patients with a higher WSS value, we may recommend carotid endarterectomy for treatment option for the consideration of the unstable plaque. However, this needs to be further confirmed. The complex blood flow such as vortex and turbulent flows was detected in the carotid stenosis. This finding is consistent with previous literature reports: the presence of high and low WSS values is usually accompanied by complex flow conditions (16, 31).

### Limitations

Several limitations exist in our study. As the inclusion criteria of our study were patients planning to undergo carotid artery stenting or carotid endarterectomy, the number of patients with mild carotid stenosis is limited. Second, although DSA can accurately provide the exact location of carotid stenosis and can clearly show the extent and severity of the disease, it still has several limitations: invasive, complicated, and relatively expensive, and DSA is limited in the display of characteristics of plaque. Third, the V flow measurements and WSS value at the opposite site of the carotid artery were not analyzed. Finally, all measurements were performed by one radiologist; further study may focus on interobserver consistency in the future.

## Conclusion

V flow technology is capable of outlining detailed depiction and visual quantification of complex flow behavior in moderate and severe carotid stenoses, which will provide a comprehensive evaluation of carotid stenosis. In addition, the WSS value will reflect the hemodynamic changes of blood viscosity and flow velocity. With the ability to obtain the WSS value easily, V flow can visually and quantitatively evaluate hemodynamic changes of the carotid stenosis. The hemodynamic changes detected by V flow of the carotid stenosis might be a potential non-invasive imaging biomarker for assessing the degree of carotid stenosis.

## Data Availability Statement

The raw data supporting the conclusions of this article will be made available by the authors, without undue reservation.

## Ethics Statement

The studies involving human participants were reviewed and approved by Ethics Committee of Zhongshan Hospital, Fudan University. The patients/participants provided their written informed consent to participate in this study.

## Author Contributions

W-PW and YD: study design and supervision. YQ and FM: ultrasound image acquirement and guidance. QZ, DY, and KC: statistical analysis. SS, DZ, XT, and LY: clinical information collection. All authors contributed to and agreed on the content of the manuscript. Each author participated sufficiently in the paper and approved the manuscript for submission.

## Conflict of Interest

The authors declare that the research was conducted in the absence of any commercial or financial relationships that could be construed as a potential conflict of interest.

## References

[1] Saxena A Ng EYK Lim ST. Imaging modalities to diagnose carotid artery stenosis: progress and prospect. Biomed Eng Online. (2019) 18:66. 10.1186/s12938-019-0685-7 31138235 PMC6537161

[2] Alagoz AN Acar BA Acar T Karacan A Demiryurek BE. Relationship between carotid stenosis and infarct volume in ischemic stroke patients. Med Sci Monit. (2016) 22:4954–9. 10.12659/MSM.898112 27984560 PMC5189723

[3] Cronenwett JL Johnston W. Rutherford's Vascular Surgery. 8th ed. Philadelphia: Saunders (2014).

[4] Eckstein HH Kuhnl A Dorfler A Kopp IB Lawall H Ringleb PA. The diagnosis, treatment and follow-up of extracranial carotid stenosis. Dtsch Arztebl Int. (2013) 110:468–76. 10.3238/arztebl.2013.0468 23964303 PMC3722642

[5] Chappell FM Wardlaw JM Young GR Gillard JH Roditi GH Yip B. Carotid artery stenosis: accuracy of noninvasive tests–individual patient data meta-analysis. Radiology. (2009) 251:493–502. 10.1148/radiol.2512080284 19276319

[6] Hung OY Brown AJ Ahn SG Veneziani A Giddens DP Samady H. Association of wall shear stress with coronary plaque progression and transformation. Interv Cardiol Clin. (2015) 4:491–502. 10.1016/j.iccl.2015.06.009 28581935

[7] Zhang X Yao ZQ Karuna T He XY Wang XM Li XF. The role of wall shear stress in the parent artery as an independent variable in the formation status of anterior communicating artery aneurysms. Eur Radiol. (2019) 29:689–98. 10.1007/s00330-018-5624-7 30019140

[8] Malek AM Alper SL Izumo S. Hemodynamic shear stress and its role in atherosclerosis. JAMA. (1999) 282:2035–42. 10.1001/jama.282.21.2035 10591386

[9] Saho T Onishi H. [Quantitative analysis of wall shear stress for human carotid bifurcation at cardiac phases by the use of phase contrast cine magnetic resonance imaging: computational fluid dynamics study]. Nihon Hoshasen Gijutsu Gakkai Zasshi. (2015) 71:1157–64. 10.6009/jjrt.2015_JSRT_71.12.1157 26685826

[10] Eshtehardi P Brown AJ Bhargava A Costopoulos C Hung OY Corban MT. High wall shear stress and high-risk plaque: an emerging concept. Int J Cardiovasc Imaging. (2017) 33:1089–99. 10.1007/s10554-016-1055-1 28074425 PMC5496586

[11] Slager CJ Wentzel JJ Gijsen FJ Schuurbiers JC van der Wal AC van der Steen AF. The role of shear stress in the generation of rupture-prone vulnerable plaques. Nat Clin Pract Cardiovasc Med. (2005) 2:401–7. 10.1038/ncpcardio0274 16119702

[12] Efstathopoulos EP Patatoukas G Pantos I Benekos O Katritsis D Kelekis NL. Wall shear stress calculation in ascending aorta using phase contrast magnetic resonance imaging. Investigating effective ways to calculate it in clinical practice. Phys Med. (2008) 24:175–81. 10.1016/j.ejmp.2008.01.004 18289907

[13] Yiu BY Lai SS Yu AC. Vector projectile imaging: time-resolved dynamic visualization of complex flow patterns. Ultrasound Med Biol. (2014) 40:2295–309. 10.1016/j.ultrasmedbio.2014.03.014 24972498

[14] Goddi A Bortolotto C Fiorina I Raciti MV Fanizza M Turpini E. High-frame rate vector flow imaging of the carotid bifurcation. Insights Imaging. (2017) 8:319–28. 10.1007/s13244-017-0554-5 28500487 PMC5438320

[15] North American Symptomatic Carotid Endarterectomy Trial C Barnett HJM Taylor DW Haynes RB Sackett DL Peerless SJ. Beneficial effect of carotid endarterectomy in symptomatic patients with high-grade carotid stenosis. N Engl J Med. (1991) 325:445–53. 10.1056/NEJM199108153250701 1852179

[16] Du Y Goddi A Bortolotto C Shen Y Dell'Era A Calliada F. Wall shear stress measurements based on ultrasound vector flow imaging: theoretical studies and clinical examples. J Ultrasound Med. (2020) 39:1649–64. 10.1002/jum.15253 32124997 PMC7497026

[17] Pedersen MM Pihl MJ Haugaard P Hansen KL Lange T Lonn L. Novel flow quantification of the carotid bulb and the common carotid artery with vector flow ultrasound. Ultrasound Med Biol. (2014) 40:2700–6. 10.1016/j.ultrasmedbio.2014.06.001 25218449

[18] Saito K Abe S Kumamoto M Uchihara Y Tanaka A Sugie K. Blood flow visualization and wall shear stress measurement of carotid arteries using vascular vector flow mapping. Ultrasound Med Biol. (2020) 46:2692–9. 10.1016/j.ultrasmedbio.2020.06.018 32753289

[19] Qiu Y Yang D Zhang Q Chen K Dong Y Wang WP. V Flow technology in measurement of wall shear stress of common carotid arteries in healthy adults: feasibility and normal values. Clin Hemorheol Microcirc. (2020) 74:453–62. 10.3233/CH-190719 31683473

[20] Goddi A Fanizza M Bortolotto C Raciti MV Fiorina I He X. Vector flow imaging techniques: an innovative ultrasonographic technique for the study of blood flow. J Clin Ultrasound. (2017) 45:582–8. 10.1002/jcu.22519 28734035

[21] Chatzizisis YS Coskun AU Jonas M Edelman ER Feldman CL Stone PH. Role of endothelial shear stress in the natural history of coronary atherosclerosis and vascular remodeling: molecular, cellular, and vascular behavior. J Am Coll Cardiol. (2007) 49:2379–93. 10.1016/j.jacc.2007.02.059 17599600

[22] Giddens DP Zarins CK Glagov S. The role of fluid mechanics in the localization and detection of atherosclerosis. J Biomech Eng. (1993) 115:588–94. 10.1115/1.2895545 8302046

[23] Goddi A Bortolotto C Raciti MV Fiorina I Aiani L Magistretti G. High-frame rate vector flow imaging of the carotid bifurcation in healthy adults: comparison with color doppler imaging. J Ultrasound Med. (2018) 37:2263–75. 10.1002/jum.14579 29574932

[24] Du Y Shen Y Yiu B. High frame rate vector flow imaging: development as a new diagnostic mode on a clinical scanner. In: Proceedings of the IEEE International Ultrasonics Symposium. Kobe (2018). p. 1–4.

[25] Bechsgaard T Hansen KL Brandt AH Holbek S Forman JL Strandberg C. Vector and doppler ultrasound velocities evaluated in a flow phantom and the femoropopliteal vein. Ultrasound Med Biol. (2017) 43:2477–87. 10.1016/j.ultrasmedbio.2017.06.020 28750944

[26] Fukumoto Y Hiro T Fujii T Hashimoto G Fujimura T Yamada J. Localized elevation of shear stress is related to coronary plaque rupture: a 3-dimensional intravascular ultrasound study with in-vivo color mapping of shear stress distribution. J Am Coll Cardiol. (2008) 51:645–50. 10.1016/j.jacc.2007.10.030 18261684

[27] Song ZZ Zhang YM. Contrast-enhanced ultrasound imaging of the vasa vasorum of carotid artery plaque. World J Radiol. (2015) 7:131–3. 10.4329/wjr.v7.i6.131 26120382 PMC4473306

[28] Eshtehardi P McDaniel MC Suo J Dhawan SS Timmins LH Binongo JN. Association of coronary wall shear stress with atherosclerotic plaque burden, composition, and distribution in patients with coronary artery disease. J Am Heart Assoc. (2012) 1:e002543. 10.1161/JAHA.112.002543 23130168 PMC3487351

[29] Lovett JK Rothwell PM. Site of carotid plaque ulceration in relation to direction of blood flow: an angiographic and pathological study. Cerebrovasc Dis. (2003) 16:369–75. 10.1159/000072559 13130178

[30] Khangure SR Benhabib H Machnowska M Fox AJ Gronlund C Herod W. Carotid near-occlusion frequently has high peak systolic velocity on Doppler ultrasound. Neuroradiology. (2018) 60:17–25. 10.1007/s00234-017-1938-4 29177789

[31] White CR Frangos JA. The shear stress of it all: the cell membrane and mechanochemical transduction. Philos Trans R Soc Lond B Biol Sci. (2007) 362:1459–67. 10.1098/rstb.2007.2128 17569643 PMC2440408

